# A rapid and naked-eye methicillin resistant *Staphylococcus aureus* screening method based on CRISPR/Cas12a and hybridization chain reaction

**DOI:** 10.3389/fmicb.2025.1592153

**Published:** 2025-07-16

**Authors:** Yayun Jiang, Zongyao Chen, Xiao Liu, Qi Xin, Dengchao Wang, Caixia Ji, Youwei Li, Gang Mai

**Affiliations:** ^1^Digestive Diseases Center, Deyang People’s Hospital, Chengdu University of Traditional Chinese Medicine, Deyang, Sichuan, China; ^2^Department of Clinical Laboratory, Deyang People’s Hospital, Chengdu University of Traditional Chinese Medicine, Deyang, Sichuan, China; ^3^Department of General Surgery (Hepatopancreatobiliary Surgery), Deyang People’s Hospital, Chengdu University of Traditional Chinese Medicine, Deyang, Sichuan, China

**Keywords:** naked-eye detection, rapid, MRSA, CRISPR/Cas12a, HCR

## Abstract

Methicillin-resistant *Staphylococcus aureus* (MRSA), a widely drug-resistant bacterium, poses a significant threat to global health. Current culturing and nucleic acid detection methods are time-consuming and require complex instruments, which do not meet the detection needs. Herein, we developed a rapid and visual MRSA detection method (MCFHCR) using ssDNA-functionalized magnetic beads as a trigger chain combined with the trans-cleavage activity of the Cas12a protein and fluorescence signal amplification of the hybridization chain reaction (HCR). MCFHCR is a signal-off platform for the detection of MRSA. In the absence of DNA targets, the trans-cleavage activity of Cas12a is inactivated, allowing HCR to proceed and form long double-stranded DNA, resulting in an increased fluorescent signal. In the presence of the DNA targets, the trans-cleavage activity of CRISPR/Cas12a is activated to cleave the trigger strand, failing HCR and leading to a decrease in the fluorescence signal. Combined with RPA, MCFHCR was completed within 35 min, achieving a limit of detection (LOD) of five copies/μL for *mecA* DNA and 8 CFU/mL for MRSA. In detecting clinical strains, MCFHCR demonstrated comparable performance to qPCR and drug sensitivity testing. Therefore, with its simple, rapid operation and convenient signal acquisition, MCFHCR shows significant practical applicability in detecting MRSA.

## Introduction

Infections caused by antibiotic-resistant bacteria have limited treatment options, leading to significant increases in morbidity and mortality and posing an important threat to human health and global public health (Jansen et al., 2018; Kuehn, 2020). Effective measures to control antimicrobial resistance include timely testing, real-time monitoring, and limiting over-the-counter antibiotics, with efficient testing being a critical factor (Balayan et al., 2020). Methicillin-resistant *Staphylococcus aureus* (MRSA), the first reported “superbug” is an important pathogen of nosocomial infections and is associated with a high death toll from antimicrobial-resistant infections across the globe (Lakhundi and Zhang, 2018; Hu et al., 2018). MRSA can cause a wide range of infections in humans, with the skin and soft tissues being the most commonly affected sites. In severe cases, MRSA infections may lead to bacteremia, pneumonia, endocarditis, and toxic shock (Kavanagh et al., 2014; Kim et al., 2018). MRSA infection directly causes more than 120,000 deaths each year, ranking first among all drug-resistant bacteria (Ikuta et al., 2022). Therefore, a rapid and sensitive MRSA detection method is urgently needed to control MRSA infection.

The standard methods for phenotypic detection of MRSA recommended by the Clinical and Laboratory Standards Institute (CLSI) are agar dilution, disk diffusion, and broth microdilution (Wiegand et al., 2008; CLSI, 2018). These methods are cumbersome and time-consuming, increasing diagnosis time and economic costs (Váradi et al., 2017). By identifying the *mecA* gene, rapid genotypic testing for MRSA enables quick recognition and effective treatment planning (Rajan et al., 2007). However, current gene detection techniques such as quantitative real-time PCR (qPCR) heavily rely on sophisticated equipment and laboratory environments, thereby constraining their applicability at the bedside and in the community (Fang and Hedin, 2003; Lee et al., 2019). Therefore, existing MRSA detection methods cannot meet clinical detection needs, especially in areas with limited medical resources.

Clustered regularly interspaced short palindromic repeats (CRISPR)/CRISPR-associated (Cas) systems are powerful gene editing technologies that have shown great application prospects in the field of rapid nucleic acid detection, which are known as the next generation of molecular detection technology (Rath et al., 2015; Makarova et al., 2011). Notably, studies have found that the CRISPR/Cas12a system has the activity of non-specific trans-cleavage single-stranded DNA (ssDNA) while recognizing and cutting target sequences (Zuo et al., 2017). This characteristic has single-base difference recognition capabilities and is used to develop particular nucleic acid detection technologies such as DETECTR (Chen et al., 2018) and HOLMES (Li et al., 2018). The sensitivity of the CRISPR/Cas12a system is still insufficient and cannot meet the needs of clinical applications by being used directly for detection. Therefore, improving sensitivity has become a hot topic in current research.

Nucleic acid isothermal amplification, an emerging technology, has broad applications in molecular detection (Chang et al., 2012; Xue et al., 2022). Hybridization chain reaction (HCR) is a powerful, enzyme-free signal amplification technology based on DNA strand displacement (Dirks and Pierce, 2004; Duan et al., 2020). HCR operates at a constant temperature and produces functional nucleic acids with alternating copolymers through hybridization reactions (Xu et al., 2015). Each initiator can trigger an HCR, forming an ultra-long hybrid DNA chain and amplifying the signal of the target molecule. Modifying the fluorescent group on the hybridized DNA chain allows for easy signal output (Hou et al., 2015; Wang et al., 2020).

In MCFHCR, two novel stem-loop structures, H1 and H2, composed of one loop and one stack, were designed. H1 is labeled with a fluorophore and a quencher, serving as switches to initiate fluorescence signal. In the stem-loop configuration, H1 remains non-fluorescent. Upon initiation of the HCR reaction, H1 unfolds, leading to the release of fluorescence signal. When no target DNA exists, the CRISPR/Cas12a remains inactivated, and MNPs-ssDNA remains intact. MNPs-ssDNA can be used as an initiator strand to activate the HCR reaction. Then, the hairpin structure of H1 was opened, and after cascade amplification, it produced significant fluorescence on the surface of magnetic beads. The cascade amplification of HCR allows the magnetic beads surface to emit fluorescence, making it visible to the naked eye under UV light. When the target gene is present, fluorescence is quenched, establishing a signal-off detection strategy. MCFHCR enables rapid and convenient operation without the need for complex instrumentation. Combined with RPA (Recombinase Polymerase Amplification), an isothermal amplification technology, the MCFHCR method had a satisfactory detection limit of 5 copies/μL for *mecA* DNA and 8 CFU/mL for MRSA. In addition, the practical application of RPA-MCFHCR was verified by the *S. aureus* isolated from clinical samples, yielding satisfactory results.

## Experimental section

### Materials and reagents

Streptavidin-coated magnetic beads were obtained from Beaver Biotechnology Ltd. (Jiangsu, China). An RPA nucleic acid amplification kit (RAAFAST) was purchased from Qitian Gene Biological Co., Ltd. (Jiangsu, China). Ultra GelRed dye was purchased from Nanjing Vazyme Biotechnology (Nanjing, China). Ethylenediaminetetraacetic acid tetrasodium salt hydrate (EDTA, 95%), sodium phosphate dibasic dodecahydrate (99%), tris (hydroxymethyl) aminomethane (99%), sodium chloride (99%), and ammonium persulfate (98%) were sourced from Aladdin Biochemical Technology Co., Ltd. (China). LbCas12a and 10 × NEBuffer 2.1 were purchased from New England Biolabs Inc. (United States). Oligonucleotide sequences (Supplementary Table S1), Primers, crRNA (Supplementary Table S2), lysostaphin, and fluorescence quantitative PCR reagents (qPCR) were acquired from Sangon Biotechnology Co., Ltd. (China).

### Preparation of MNPs-ssDNA

Avidin-modified magnetic beads were vortexed thoroughly for 30 s and washed thrice with the 2 × BW Buffer using a magnetic separator to retain the beads. Then, 30 μL of biotin-modified DNA (10 μM) was added to the avidin-modified magnetic beads, which were incubated at room temperature with shaking for 30 min. The magnetic beads were washed with 1 × BW Buffer three times and resuspended in 80 μL of 1 × BW to obtain 5 mg/mL of MNPs-ssDNA probe.

### HCR reaction

According to the principle of Watson-Crick base pairing, the initiator strand H0 and two metastable auxiliary DNA hairpins (H1 and H2) were designed for the HCR reaction. UNPACK software was used to analyze the secondary structures of H1/H2 and the standard Gibbs free energy changes of the HCR reaction, ensuring the specificity and sensitivity. The 10 μM of H1 and H2 were incubated at 95°C for 10 min and naturally cooled to room temperature to form a hairpin of H1 and H2. Afterward, 1 μL of 10 μM H0 was mixed with 10 μL of 10 μM H1/H2 and incubated at 37°C with shaking for 10 min. Finally, the feasibility of HCR was verified by 12% polyacrylamide gel electrophoresis.

### Measurement of the MCFHCR

A mixture of 4 μL of 100 nM Cas12a, 20 μL of 200 nM crRNA, 1 μL of 50 nM MNPs-ssDNA, 2 μL of DNA target, and 3 μL of 10 × NEB buffer 2.1 was prepared and incubated at 37°C for 30 min. The magnetic beads were washed three times with 1 × BW Buffer, and then the HCR reaction was performed on the surface of the magnetic beads. Fluorescence was visualized under UV light and then transferred to a 384-well plate for fluorescence spectrum measurement using a microplate reader.

### Bacterial culture and genomic DNA extraction

The strains were inoculated on blood agar plates and incubated at 35°C overnight. A single colony was then selected and inoculated in an LB liquid medium for enrichment at 35°C with shaking at 250 rpm. The enriched bacteria were collected by centrifugation and washed twice with PBS. The bacterial concentration was estimated by adjusting the optical density of the suspension to an absorbance of 1.0 at 600 nm. The bacterial suspension was then serially diluted, and the exact concentration was measured using the plate count method (Supplementary Figure S1). The genomic DNA extraction was performed using the heated lysis method (100°C for 5 min). The heated bacterial suspension was centrifuged to remove the sediments, and the genomic DNA remained in the supernatant. All DNA templates were stored at −20°C for further use.

### Clinical strain detection

All clinical strains were obtained from Deyang People’s Hospital, and the Medical Research Ethics Committee approved the study. The method for extracting bacterial genomic DNA was the same as described in the previous section. The MIC method verified the methicillin susceptibility of all *S. aureus* strains by the Vitek 2 system. Additionally, the expression of *mecA* in each strain was verified by qPCR. Following the manufacturer’s protocol, the qPCR assay was performed using a qPCR kit (Sangon, China) and SLAN-96P real-time PCR system (Hongshi, China). The RPA reaction was conducted in a constant temperature metal bath at 37°C for 10 min. Specifically, 50 μL of RPA reaction mixture contained 25 μL of buffer V, 5 μL of magnesium acetate, 11 μL of purified water, 2 μL of forward primer (10 μM), 2 μL of reverse primer (10 μM), and 5 μL of DNA template.

## Results and discussion

### Principle of the MCFHCR method

**Figure 1:**
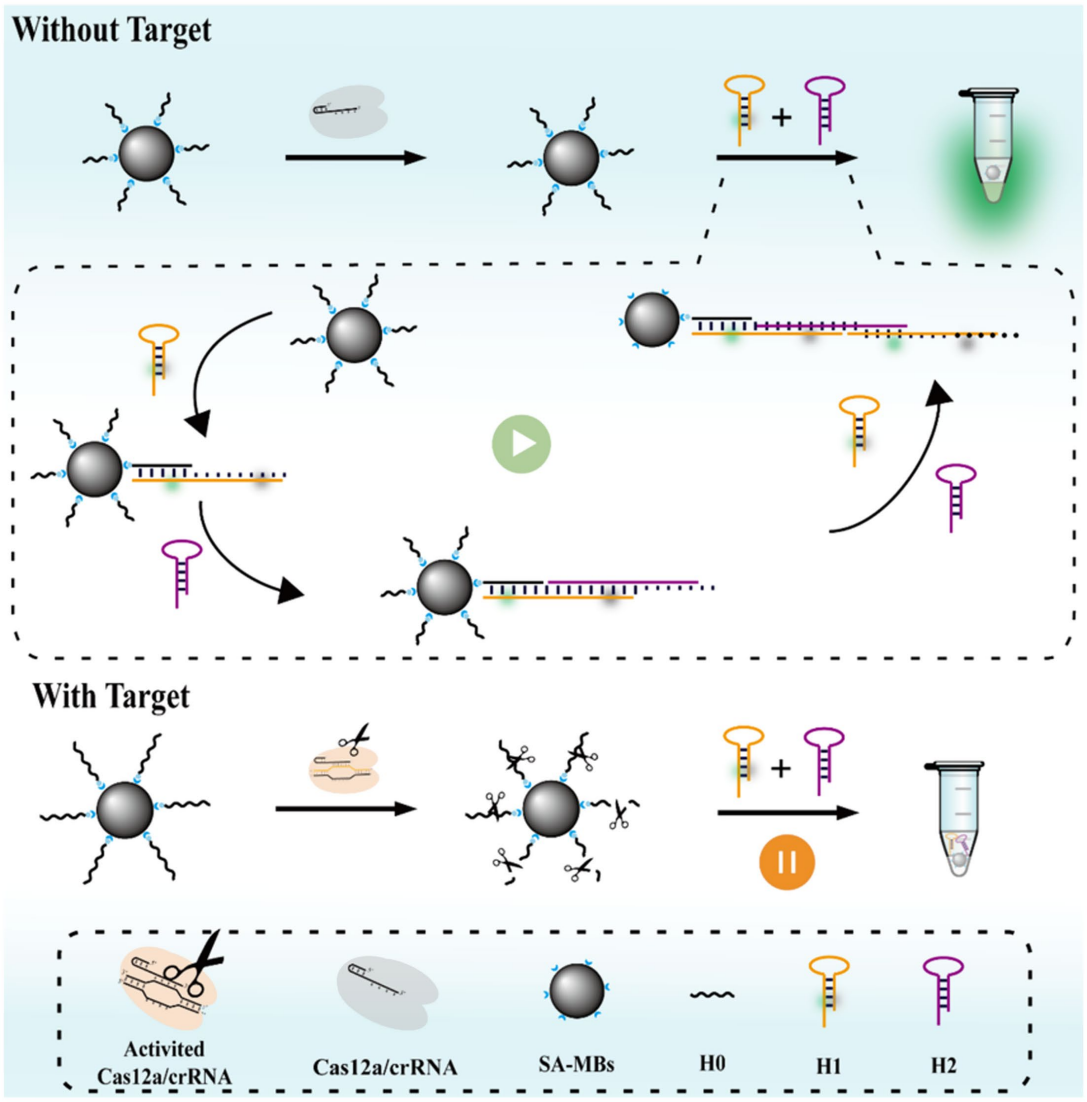
Schematic illustration of the MCFHCR method.

As illustrated in Figure 1, MCFHCR mainly consists of two processes: target DNA-triggered trans-cleavage activity of the CRISPR/Cas12a system and HCR-mediated fluorescence amplification on the surface of magnetic beads. The 5′ biotin-modified single-stranded DNA is designed to construct the MNPs-ssDNA signal probe, achieved by attaching the avidin-modified magnetic beads to the ssDNA. Two new hairpins, H1 and H2, were designed. H1 is labeled with a fluorophore and a quencher, acting as switches to initiate fluorescence signaling. Without target DNA, the CRISPR/Cas12a remains inactivated, and MNPs-ssDNA remains intact. MNPs-ssDNA can serve as an initiator strand to activate the HCR reaction. Then, the hairpin structure of H1 was opened after cascade amplification. After a series of hybridization reactions, a double-stranded DNA with the H1/H2 sequence is finally formed. The alteration of the hairpin structure causes the separation of the fluorophore and quencher, resulting in fluorescence emission. In the presence of target DNA, the MNPs-ssDNA were indiscriminately cleaved, and there was no corresponding signal output.

### Validation of the MCFHCR method

**Figure 2:**
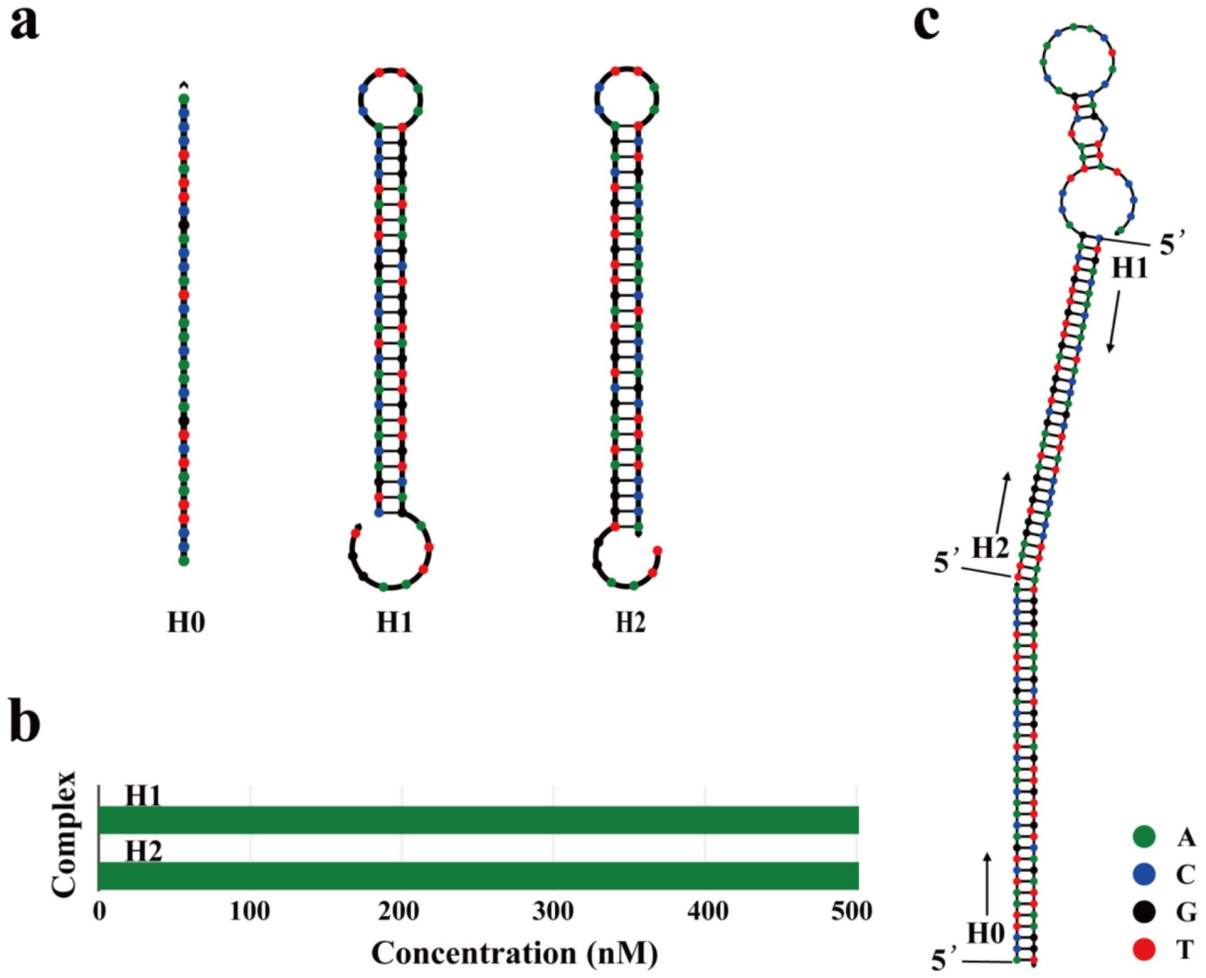
Design of the HCR sequences. **(a)** UNPACK software online is used to predict the secondary structure of H0, H1, and H2. The calculated standard Gibbs energy changes of H0, H1, and H2 are 0.00 Kcal/mol, −31.17 Kcal/mol, and −49.44 Kcal/mol, respectively. **(b)** Equilibrium complex concentrations of H1 and H2 at 500 nM using UNPACK software online. The horizontal axis indicates the concentrations of different complexes, and the vertical axis shows their corresponding names. The green bars represent the predicted concentrations of each complex at thermodynamic equilibrium. **(c)** Predicted the secondary structure of 500 nM H0, H1, and H2 at 37°C using UNPACK software online.

Firstly, the trigger chain (H0) and hairpin structures (H1 and H2) of the hybridization reaction were designed according to the principle of base-pairing and self-assembly characteristics of nucleic acids (Supplementary Table S1). The secondary structure and Gibbs free energy were predicted online by NUPACK software. H0 showed no secondary structure and had a Gibbs free energy of 0 kcal/mol. H1 and H2 each formed a stem-loop structure composed of one loop and one stack, and the Gibbs free energy values were −31.17 and −49.44 kcal/mol, respectively (Figure 2a). In addition, the hybridization of 500 nM H0, H1, and H2 at 37°C was predicted. As shown in Figure 2b, the two single-stranded DNAs did not have obvious complementary pairing and existed in a single-stranded free state without the H0. Finally, the hybridization of 500 nM H0, H1, and H2 was predicted using NUPACK software. As shown in Figure 2c, the H0 opened the hairpin DNA structures H1 and H2 in sequence and propagated a chain reaction of the amplification process.

**Figure 3:**
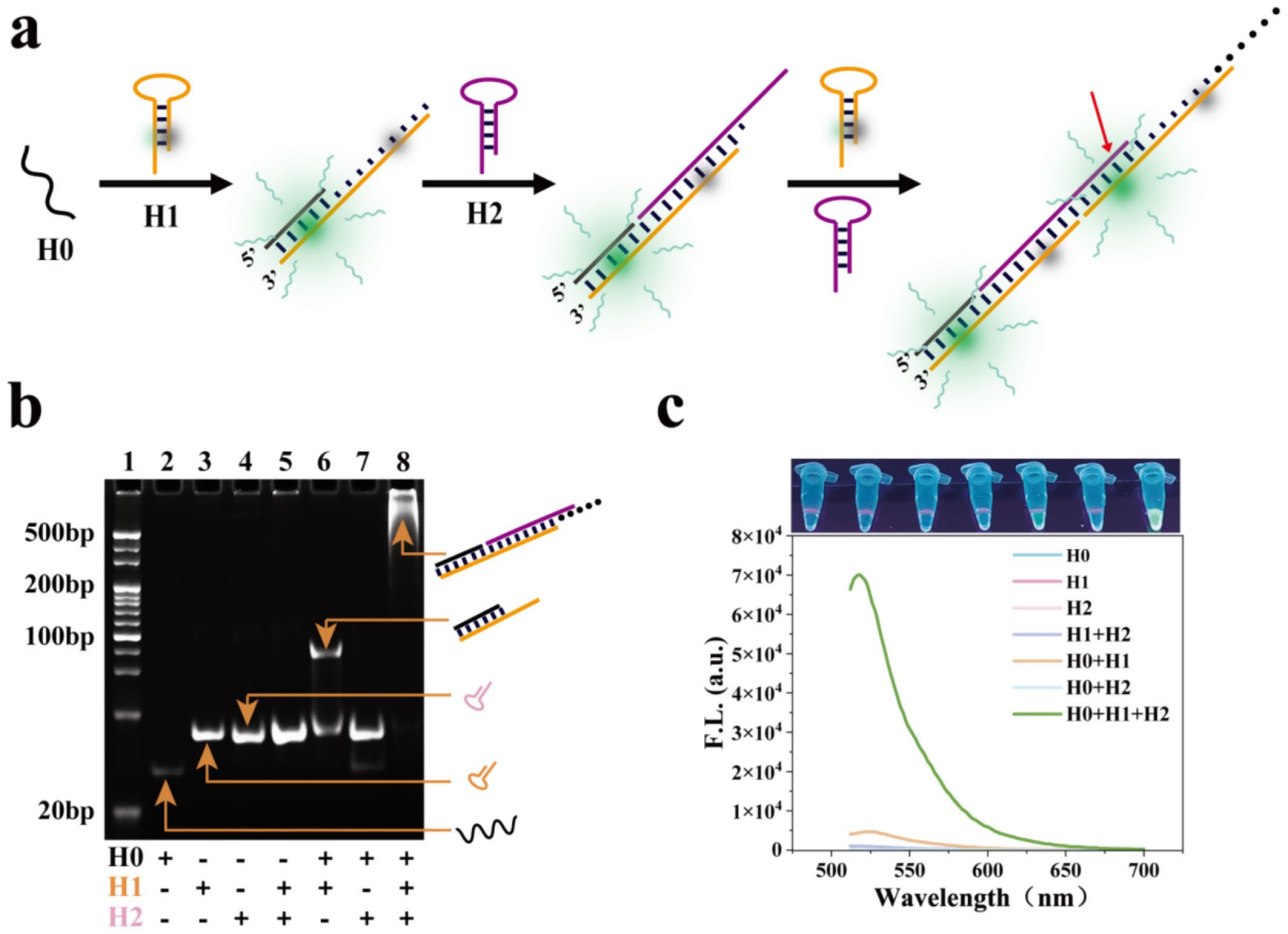
Feasibility analysis of HCR sequences. **(a)** Schematic illustration of the DNA hybridization of fluorescent HCR sequences. The red arrow indicates that the fluorophore is located in the middle of the two quenchers. **(b)** Verification of HCR sequences’ feasibility using 12% polyacrylamide gel electrophoresis. Lane 1: markers. Lane 2: H0. Lane 3: H1. Lane 4: H2. Lane 5: H, H2. Lane 6: H0, H1. Lane 7: H0, H2. Lane 8: H0, H1, H2. **(c)** Fluorescence spectra demonstrate the feasibility of the HCR sequence (Top: the photograph of detection results under UV light).

A series of experiments were conducted to validate each component of the MCFHCR method. First, the feasibility of HCR sequences was verified using 12% polyacrylamide gel electrophoresis. As shown in Figure 3b, no new bands appeared in lane 5 (H1 and H2), indicating that the structures of H1 and H2 were stable. A new band in lane 6 demonstrates that H0 can open the hairpin structure of H1, forming a hybridized chain. Large fragment bands were observed in the H0, H1, and H2 groups (lane 8), indicating that HCR achieved full hybridization and confirming the feasibility of the HCR reaction in lane 8. Subsequently, we verified the fluorescence signal amplification effect of HCR (Figure 3a). As the hairpin structure is formed, the fluorophore and quencher labeled on H1 are in the stem-closed structure, and the quencher suppresses fluorescence from the fluorophore. When the HCR reaction is initiated, the crossing-opening of the hairpins H1 and H2 forms a repeated double-stranded DNA labeled with the fluorophore and quencher alternately. In this situation, the distance between the fluorophores and the quenchers increases, and the fluorophore emits a fluorescence signal. In addition, we designed the fluorophore to be at the farthest position from the two adjacent quenchers to maximize the fluorescence signal. The results showed that H1 and H2 hairpin structures were stable, H0 could open hairpin H1, and HCR produced effective fluorescence amplification (Figure 3c).

**Figure 4:**
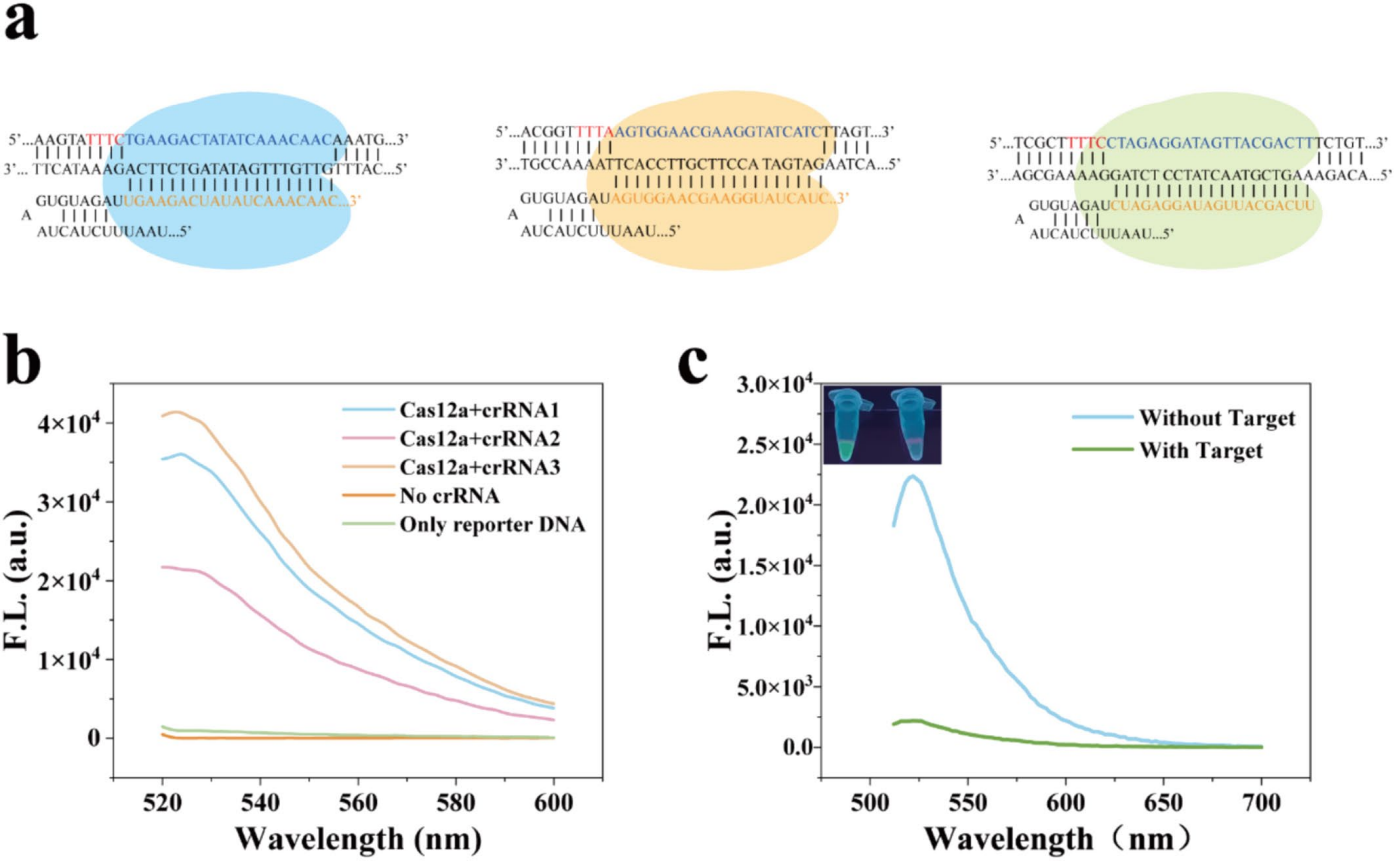
Feasibility analysis of the MCFHCR method. **(a)** Schematic illustration of the crRNA sequence. **(b)** Fluorescence spectra of ssDNA-FQ cleaved by different crRNA sequences in the CRISPR/Cas12a system. **(c)** Feasibility analysis of MCFHCR fluorescence visualization detection.

To enhance the trans-cleavage activity of Cas12a, we designed three crRNAs (Figure 4a). Detailed sequence information of crRNA is provided in Supplementary Table S2. A Cas12a-mediated cleavage assay was conducted, and fluorescence spectroscopy indicated that crRNA3 exhibited the highest cleavage activity, so crRNA3 was used in further experiments (Figure 4b; Jiang et al., 2023). Finally, the entire feasibility of visual detection for the MCFHCR method was verified. As shown in Figure 4c, in the absence of target DNA, the MNPs-ssDNA remains intact and acts as a trigger chain to activate the HCR reaction, resulting in a fluorescence signal. In the presence of target DNA, MNPs-ssDNA were indiscriminately cleaved, resulting in no corresponding signal output.

### Optimization of experimental conditions

**Figure 5:**
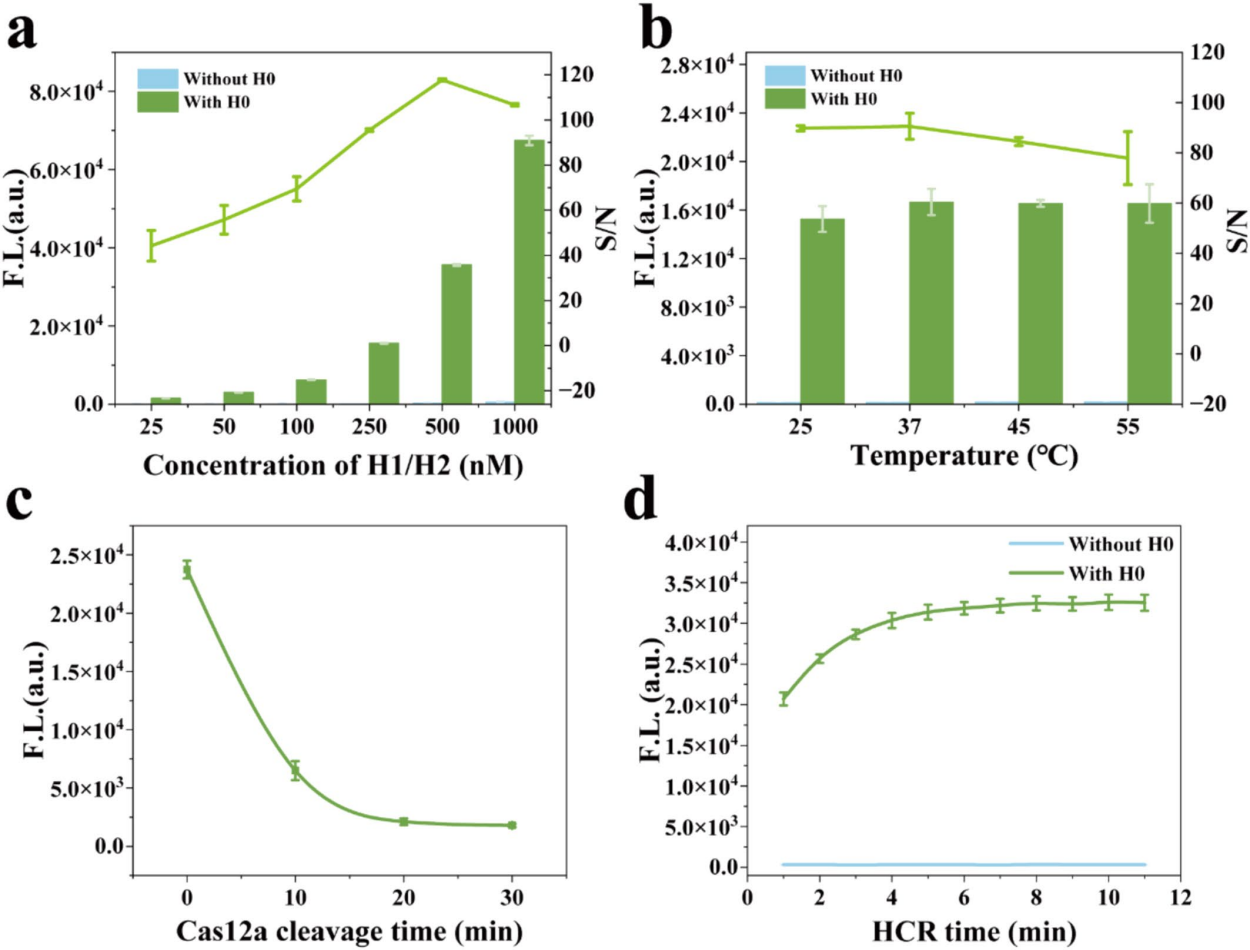
Optimization of the reaction parameters. **(a)** The H1/H2 concentrations of 25, 50, 100, 250, 500, and 1,000 nM were optimized by the S/N ratio of the fluorescence signal. **(b)** The temperatures of 25, 37, 45, and 55°C were optimized by the S/N ratio of the fluorescence signal. **(c)** The fluorescence signal of the CRISPR/Cas12a reaction at 0, 10, 20, and 30 min. **(d)** The fluorescence signal of HCR hybridization from 0 to 11 min. Error bars represent mean ± SD, where *n* = 3 replicates.

To achieve excellent detection performance of the MCFHCR method, experimental conditions associated with the HCR and CRISPR/Cas12a systems were optimized. The concentration of hairpin probes (H1 and H2) is a key factor influencing HCR efficiency. As shown in Figure 5a, the S/N ratio increases and decreases as the hairpin concentration rises, peaking at 500 nM. Therefore, we selected 500 nM of H1/H2 for subsequent experiments. Additionally, we optimized the effects of 25, 37, 45, and 55°C on the HCR reaction (Figure 5b). The S/N ratio of MCFHCR does not differ significantly at lower temperatures, which gives us a wider range of temperature options. In addition, we optimized the pH and reaction temperature of the HCR reaction. As shown in Supplementary Figure S2, the pH and temperature had no obvious effect on the HCR reaction. The CRISPR/Cas12a cleavage and HCR reaction times were optimized to shorten the detection time. Figure 5c illustrates the fluorescence signal changes during CRISPR/Cas12a cleavage from 0 to 30 min. The fluorescence intensity gradually decreases and eventually reaches a plateau, with no significant changes observed thereafter at 15 min. Therefore, we chose CRISPR/Cas12a cleavage for 15 min for subsequent experiments. The HCR time is shown in Figure 5d. Hairpin H1 rapidly opens on the surface of the magnetic beads and reaches a plateau within 5 min. Based on this result, a 5-min reaction time was selected for subsequent experiments, significantly reducing the overall detection time.

### Analytical performance of the MCFHCR for MRSA

To determine the threshold (TH) of the MCFHCR platform, we conducted 12 repeated experiments using negative samples (ATCC 29213) (Supplementary Figure S3). The threshold was calculated using the formula TH = X − 3SD, where X is the mean signal intensity and SD is the corresponding standard deviation. Specifically, TH = 24671.91–3 × 999.01 = 21674.88. Therefore, the threshold value of 21674.88 is used to distinguish between positive and negative results. To evaluate the analytical performance of the MCFHCR detection platform, we first tested its nucleic acid detection capability at various concentrations of target DNA, ranging from 0 to 5 × 10^6^ copies/μL. As shown in Supplementary Figure S4, when the concentration of the target gene is 5 × 10^5^ copies/μL, the fluorescence intensity is lower than the threshold line. Therefore, MCFHCR can detect 5 × 10^5^ copies/μL of *mecA* without other amplification.

**Figure 6:**
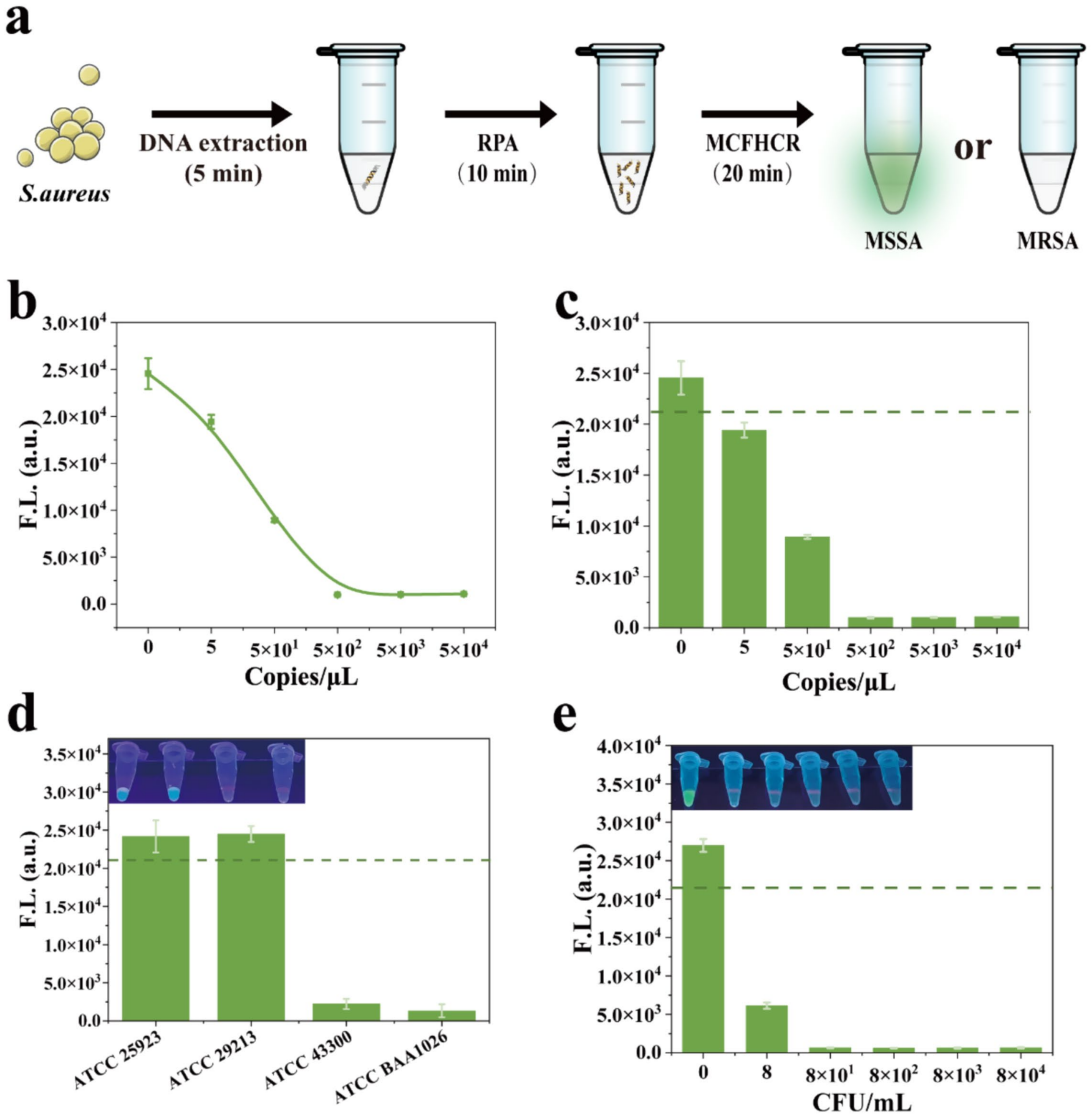
Analytical performance of the RPA-MCFHCR method. **(a)** Schematic illustration of the RPA-MCFHCR method for the detection of MRSA. **(b)** Fluorescence intensity changes of the RPA-MCFHCR system in response to serial dilutions of target DNA ranging from 0 to 5 × 10^4^ copies/μL. **(c)** Bar graph of fluorescence signals under the same target DNA concentrations. **(d)** Specificity analysis of the RPA-MCFHCR method by measurement of ATCC 25923 (MSSA), ATCC 29213 (MSSA), ATCC 43300 (MRSA), ATCC BAA1026 (MRSA). **(e)** Sensitivity analysis of the RPA-MCFHCR method by different concentrations of MRSA, ranging from 0 to 8 × 10^4^ CFU/mL. Error bars represent mean ± SD, where *n* = 3 replicates. The visual observation results are listed above the corresponding results. The dashed line indicates the threshold value distinguishing positive from negative samples.

To shorten the detection time, enhance sensitivity, and advance MCFHCR’s clinical application, we combine it with RPA. The *mecA* targets were rapidly amplified by RPA, thereby improving the performance of MCFHCR (Figure 6a). We then tested the nucleic acid detection capability of RPA-MCFHCR at various concentrations of target DNA, ranging from 0 to 5 × 10^4^ copies/μL. As shown in Figure 6b, the fluorescence signal gradually decreased with increasing target concentration, exhibiting a typical signal-off response pattern. This trend was further illustrated in the bar graph (Figure 6c). Based on the defined threshold line, the DNA limit of detection (LOD) of RPA-MCFHCR was 5 copies/μL. The sequence-specific recognition by the CRISPR/Cas12a system and the selective HCR sequences ensured MCFHCR’s specificity in detecting MRSA. The specificity of the MCFHCR method for MRSA was investigated with two methicillin-sensitive standard strains (ATCC 29213, ATCC 25923) and two methicillin-resistant standard strains (ATCC 43300 and ATCC BAA1026). As shown in Figure 6d, the fluorescence of MSSA was significantly higher than that of MRSA. Similar results were observed in fluorescence images under UV light. We also evaluated the sensitivity of the MCFHCR system for MRSA under optimal conditions. As the concentration of MRSA increased, fluorescence intensity rapidly decreased. A significant variation was observed when the MRSA concentration reached 8 CFU/mL compared with 0 CFU/mL, and fluorescence became undetectable as the colony count increased (Figure 6e). Thus, RPA-MCFHCR demonstrates strong detection performance for MRSA, with a detection limit (LOD) reaching 8 CFU/mL. Furthermore, we also compared the MCFHCR with other reported CRISPR/Cas12a-based methods regarding readout system, detection time, amplification or not, instrument requirement, and LOD (Table 1). The MCFHCR presents excellent performance on LOD compared to previously described methods, and offers additional advantages such as rapid operation and low dependence on equipment.

**Table 1:** Comparison of different CRISPR-based detection.

Readout system	Target	Time	Amplification	Instrument requirement	LOD	References
Lateral flow strip	*Staphylococcus aureus*	40 min	LAMP	None	670 CFU/mL	Xu et al. (2023)
Fluorescence	*Listeria monocytogenes*	45 min	RPA	UV torch	10 CFU/mL	Tian et al. (2021)
Electrochemical biosensor	*Listeria monocytogenes*	45 min	RPA	Electrochemical workstation	26 CFU/mL	Li et al. (2021)
Colorimetry	*Vibrio parahaemolyticus*	> 90 min	LAMP, DNAzyme	None	610 CFU/mL	Chen et al. (2021)
Fluorescence	*Vibrio parahaemolyticus*	90 min	PCR	Thermal cycler	100 CFU/g	Zhang et al. (2020)
Fluorescence	MRSA	35 min	RPA, HCR	UV torch	8 CFU/mL	This work

### Performance of the RPA-MCFHCR for clinical strains

**Figure 7:**
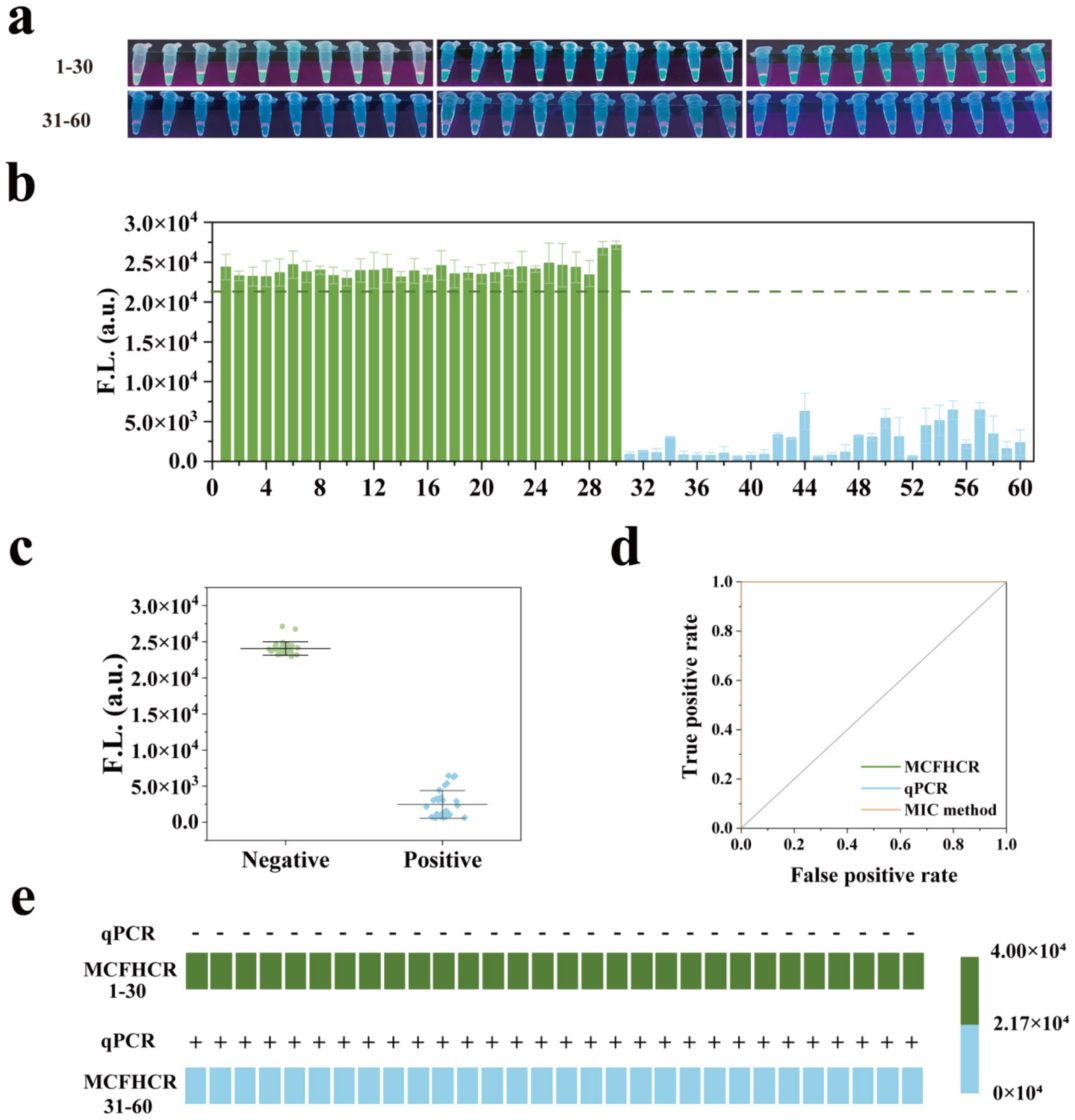
Performance of the RPA-MCFHCR method for clinical strains. **(a)** Visualized results of RPA-MCFHCR for clinical strains (1–30: MSSA; 31–60: MRSA). **(b)** The fluorescence intensity of 60 clinical strains at 522 nm by RPA-MCFHCR (1–30: MSSA; 31–60: MRSA). **(c)** Scatter plot analysis of the intensity for all positive samples and negative samples. **(d)** ROC curves of the RPA-MCFHCR, qPCR, and susceptibility testing in clinical strains for MRSA. AUC for RPA-MCFHCR, qPCR, and susceptibility testing are both 1.000. **(e)** Heat map analysis of MRSA detected by the RPA-MCFHCR and qPCR. Error bars represent mean ± SD, where *n* = 3 replicates.

To evaluate the practical performance of RPA-MCFHCR, we collected 60 clinical strains of *S. aureus* from secretions, sputum, and blood specimens, including 30 MRSA and 30 MSSA (Supplementary Table S3). Methicillin resistance in all strains was verified using the MIC method via the Vitek 2 system and regarded as the gold standard (Clinical and Laboratory Standards Institute, 2020). In the RPA-MCFHCR test, all 30 strains of MSSA exhibited clear fluorescence. In contrast, none of the MRSA strains showed fluorescence under UV light (Figure 7a). Similarly, fluorescence detection confirmed that all MSSA strains emitted significant fluorescence above the threshold. In contrast, all MRSA strains displayed significantly reduced fluorescence below the threshold (Figure 7b). The scatter plot analysis revealed a significant difference in fluorescence intensity between the two groups, with the negative group exhibiting markedly higher values than the positive group (Figure 7c). We also used qPCR to detect the presence of the *mecA* gene in all strains (Supplementary Figure S5). As shown in Figure 7e, the RPA-MCFHCR results for all 60 clinical strains of *S. aureus* are exactly as expected with results obtained using qPCR. The sensitivity and specificity of RPA-MCFHCR and qPCR measurements were derived from the ROC curves, which showed comparable area under the curve (AUC) values of both 1.00 (Figure 7d). The detection performance of RPA-MCFHCR is consistent with that of the qPCR and MIC, achieving satisfactory detection performance. Therefore, the RPA-MCFHCR method holds great potential for detecting drug-resistant bacteria in clinical applications.

## Discussion

MRSA is highly pathogenic and resistant to multiple antibiotics, posing a significant threat to global public health. Traditional culture-based drug sensitivity tests are time-consuming and do not meet clinical testing needs. The PCR method targeting *mecA* is considered the gold standard for MRSA molecular detection and is widely used in diagnosis. Many studies have demonstrated the value of PCR in detecting MRSA, but it requires specialized instruments and technicians, limiting its use in POCT (Ding et al., 2022). More researchers have begun to pay attention to developing new molecular detection methods for MRSA.

Developing a simple and rapid MRSA visual detection technology is crucial for protecting patient health and controlling hospital infections. In this study, we developed a rapid and sensitive visual fluorescence assay for MRSA, namely MCFHCR. MCFHCR consists of three main components: ssDNA-functionalized magnetic beads, the trans-cleaving function of CRISPR/Cas12a, and fluorescence signal amplification by HCR. We designed three crRNAs targeting the MRSA-specific target gene *mecA* to assess their feasibility and cutting efficiency using an ELISA reader, and identified crRNA3 as the most effective. DNA-functionalized magnetic beads were prepared using avidin-modified magnetic beads and biotin-modified DNA to initiate HCR reactions. Two new stem-loop structures, H1 and H2, were designed. H1 is labeled with a fluorophore and a quencher, serving as switches to initiate fluorescence signaling. In the presence of H0, the stem-loop structures of H1 and H2 open sequentially to form a hybrid chain and generate fluorescent signals. The feasibility of MCFHCR was demonstrated by MRSA and showed that the results can be easily read by naked eye observation.

To improve the detection performance of MCFHCR, we optimized the concentration of H1/H2, HCR reaction temperature, Cas12a cleavage time, and HCR time, respectively. Under optimal conditions, MCFHCR’s selectivity and sensitivity were evaluated. MCFHCR exhibits good selectivity and can easily distinguish MRSA from MSSA with the naked eye. By utilizing RPA amplification, CRISPR/Cas12a cleavage, and HCR amplification, MCFHCR achieves a detection limit of 5 copies/μL for *mecA* DNA and 8 CFU/mL for MRSA. Importantly, rigorous optimization of experimental conditions allowed the entire detection process to be completed within 35 min, greatly improving detection efficiency. Finally, we tested 30 MRSA and 30 MSSA clinical strains to assess RPA-MCFHCR’s performance in clinical applications. RPA-MCFHCR performed well in detecting clinical *Staphylococcus aureus* strains, with results consistent with the MIC and qPCR methods.

RPA-MCFHCR offers high sensitivity, rapidity, and visualization, making it suitable for bedside and resource-limited settings. RPA-MCFHCR uses a small amount of H0 as a promoter, reducing the substrate cleavage by Cas12a and improving sensitivity. In addition, fewer cleavage substrates shorten cleavage time, and optimizing Cas12a cleavage and HCR reaction times allows MRSA identification within 35 min. By utilizing magnetic bead separation and fluorescence signal visualization, RPA-MCFHCR enables result reading without complex instruments (Supplementary Figure S6). Overall, the RPA-MCFHCR presents excellent performance on LOD compared to previously described methods, and offers additional advantages such as rapid operation and low dependence on equipment. This study provides a simple and easy method for detecting MRSA, which can be extended to other drug-resistant bacteria based on the editability of crRNA. We envisage that our proposed RPA-MCFHCR has broad application prospects in clinical diagnosis, environmental science, and other fields.

## Data Availability

The original contributions presented in the study are included in the article/Supplementary material, further inquiries can be directed to the corresponding authors.

## References

[ref1] Balayan S. Chauhan N. Chandra R. Kuchhal N. K. Jain U. (2020). Recent advances in developing biosensing based platforms for neonatal sepsis. Biosens. Bioelectron. 169:112552. doi: 10.1016/j.bios.2020.112552, PMID: 32931992

[ref2] Chang C. C. Chen C. C. Wei S. C. Lu H. H. Liang Y. H. Lin C. W. (2012). Diagnostic devices for isothermal nucleic acid amplification. Sensors 12, 8319–8337. doi: 10.3390/s120608319, PMID: 22969402 PMC3436031

[ref3] Chen J. S. Ma E. Harrington L. B. Da Costa M. Tian X. Palefsky J. M.. (2018). CRISPR-Cas12a target binding unleashes indiscriminate single-stranded DNase activity. Science 360, 436–439. doi: 10.1126/science.aar6245, PMID: 29449511 PMC6628903

[ref4] Chen X. Wang L. He F. Chen G. Bai L. He K.. (2021). Label-free colorimetric method for detection of *Vibrio parahaemolyticus*by trimming the G-quadruplex DNAzyme with CRISPR/Cas12a. Anal. Chem. 93, 14300–14306. doi: 10.1021/acs.analchem.1c03468 34645259

[ref5] CLSI (2018) Clinical and Laboratory Standards Institute. Clinical and laboratory standards institute methods for dilution antimicrobial susceptibility tests for bacteria that grow aerobically standard, approval CDM-A.; M07 Methods dilution Antimicrob. Susceptibility Tests Bact. That Grow Aerob. 91.

[ref6] Clinical and Laboratory Standards Institute (2020). CLSI M100-ED29: 2021 performance standards for antimicrobial susceptibility testing. 30th Edn.

[ref7] Ding X. Wang H. Cui M. Cheng M. Zhao Q. Bai Y.. (2022). Development of a Real-Time Recombinase-Aided Amplification Method to Rapidly Detect Methicillin-Resistant Staphylococcus aureus. Staphylococcus aureus Microorganisms. 10:2351. doi: 10.3390/microorganisms10122351 36557604 PMC9784193

[ref8] Dirks R. M. Pierce N. A. (2004). Triggered amplification by hybridization chain reaction. Proc. Natl. Acad. Sci. USA 101, 15275–15278. doi: 10.1073/pnas.0407024101, PMID: 15492210 PMC524468

[ref9] Duan Y. Li Y. Zhang C. Chen J. Sun R. Huang Z.. (2020). The recent development of hybridization chain reaction strategies in biosensors. ACS Sens. 5, 2977–3000. doi: 10.1021/acssensors.0c01453, PMID: 32945653

[ref10] Fang H. Hedin G. (2003). Rapid screening and identification of methicillin-resistant *Staphylococcus aureus* from clinical samples by selective-broth and real-time PCR assay. J. Clin. Microbiol. 41, 2894–2899. doi: 10.1128/JCM.41.7.2894-2899.2003, PMID: 12843018 PMC165274

[ref11] Hou L. Wu X. Chen G. Yang H. Lu M. Tang D. (2015). HCR-stimulated formation of DNAzyme concatamers on gold nanoparticle for ultrasensitive impedimetric immunoassay. Biosens. Bioelectron. 68, 487–493. doi: 10.1016/j.bios.2015.01.043, PMID: 25636020

[ref12] Hu F. Zhu D. Wang F. Wang M. (2018). Current status and trends of antibacterial resistance in China. Clin. Infect. Dis. 67, S128–S134. doi: 10.1093/cid/ciy657, PMID: 30423045

[ref13] Ikuta K. S. Swetschinski L. R. Aguilar G. R. Sharara F. Mestrovic T. Gray A. P.. (2022). Global mortality associated with 33 bacterial pathogens in 2019: a systematic analysis for the global burden of disease study 2019. Lancet 400, 2221–2248. doi: 10.1016/S0140-6736(22)02185-7, PMID: 36423648 PMC9763654

[ref14] Jansen K. U. Knirsch C. Anderson A. S. (2018). The role of vaccines in preventing bacterial antimicrobial resistance. Nat. Med. 24, 10–19. doi: 10.1038/nm.4465, PMID: 29315295

[ref15] Jiang Y. Zheng C. Jin M. Zhou R. Wu Q. Huang F.. (2023). An ultrasensitive colorimetric foodborne pathogenic detection method using a CRISPR/Cas12a mediated Strand displacement/hybridization chain reaction. J. Agric. Food Chem. 71, 4193–4200. doi: 10.1021/acs.jafc.2c08888, PMID: 36812357

[ref16] Kavanagh K. T. Calderon L. E. Saman D. M. Abusalem S. K. (2014). The use of surveillance and preventative measures for methicillin-resistant *staphylococcus aureus* infections in surgical patients. Antimicrob. Resist. Infect. Control 3:18. doi: 10.1186/2047-2994-3-18, PMID: 24847437 PMC4028005

[ref17] Kim M. W. Greenfield B. K. Snyder R. E. Steinmaus C. M. Riley L. W. (2018). The association between community-associated *Staphylococcus aureus* colonization and disease: a meta-analysis. BMC Infect. Dis. 18:86. doi: 10.1186/s12879-018-2990-3, PMID: 29466953 PMC5822478

[ref18] Kuehn B. M. (2020). Alarming antimicrobial resistance trends emerge globally. JAMA 324:223. doi: 10.1001/jama.2020.11330, PMID: 32692393

[ref19] Lakhundi S. Zhang K. (2018). Methicillin-Resistant Staphylococcus aureus: Molecular Characterization, Evolution, and Epidemiology. Clin. Microbiol. 31:e00020–18. doi: 10.1128/CMR.00020-18 PMC6148192 30209034

[ref20] Lee S. H. Park S.-M. Kim B. N. Kwon O. S. Rho W.-Y. Jun B.-H. (2019). Emerging ultrafast nucleic acid amplification technologies for next-generation molecular diagnostics. Biosens. Bioelectron. 141:111448. doi: 10.1016/j.bios.2019.111448, PMID: 31252258

[ref21] Li S.-Y. Cheng Q.-X. Wang J.-M. Li X.-Y. Zhang Z.-L. Gao S.. (2018). CRISPR-Cas12a-assisted nucleic acid detection. Cell Discov. 4:20. doi: 10.1038/s41421-018-0028-z, PMID: 29707234 PMC5913299

[ref22] Li F. Ye Q. Chen M. Zhou B. Zhang J. Pang R.. (2021). An ultrasensitive CRISPR/Cas12a based electrochemical biosensor for *Listeria monocytogenes* detection. Biosens. Bioelectron. 179:113073. doi: 10.1016/j.bios.2021.113073, PMID: 33581428

[ref23] Makarova K. S. Haft D. H. Barrangou R. Brouns S. J. J. Charpentier E. Horvath P.. (2011). Evolution and classification of the CRISPR-Cas systems. Nat. Rev. Microbiol. 9, 467–477. doi: 10.1038/nrmicro2577, PMID: 21552286 PMC3380444

[ref24] Rajan L. Smyth E. Humphreys H. (2007). Screening for MRSA in ICU patients. How does PCR compare with culture? J. Infect. 55, 353–357. doi: 10.1016/j.jinf.2007.06.005, PMID: 17686525

[ref25] Rath D. Amlinger L. Rath A. Lundgren M. (2015). The CRISPR-Cas immune system: biology, mechanisms and applications. Biochimie 117, 119–128. doi: 10.1016/j.biochi.2015.03.025, PMID: 25868999

[ref26] Tian Y. Liu T. Liu C. Xu Q. Fang S. Wu Y.. (2021). An ultrasensitive and contamination-free on-site nucleic acid detection platform for *Listeria monocytogenes* based on the CRISPR-Cas12a system combined with recombinase polymerase amplification. Lwt 152:112166. doi: 10.1016/j.lwt.2021.112166

[ref27] Váradi L. Luo J. L. Hibbs D. E. Perry J. D. Anderson R. J. Orenga S.. (2017). Methods for the detection and identification of pathogenic bacteria: past, present, and future. Chem. Soc. Rev. 46, 4818–4832. doi: 10.1039/c6cs00693k, PMID: 28644499

[ref28] Wang Y. Zhao X. Zhang M. Sun X. Bai J. Peng Y.. (2020). A fluorescent amplification strategy for high-sensitive detection of 17 β-estradiol based on EXPAR and HCR. Anal. Chim. Acta 1116, 1–8. doi: 10.1016/j.aca.2020.04.010, PMID: 32389184

[ref29] Wiegand I. Hilpert K. Hancock R. E. W. (2008). Agar and broth dilution methods to determine the minimal inhibitory concentration (MIC) of antimicrobial substances. Nat. Protoc. 3, 163–175. doi: 10.1038/nprot.2007.521, PMID: 18274517

[ref30] Xu M. Gao Z. Wei Q. Chen G. Tang D. (2015). Hemin/G-quadruplex-based DNAzyme concatamers for in situ amplified impedimetric sensing of copper(II) ion coupling with DNAzyme-catalyzed precipitation strategy. Biosens. Bioelectron. 74, 1–7. doi: 10.1016/j.bios.2015.05.056, PMID: 26093122

[ref31] Xu D. Zeng H. Wu W. Liu H. Wang J. (2023). Isothermal Amplification and CRISPR/Cas12a-System-Based Assay for Rapid, Sensitive and Visual Detection of Staphylococcus aureus. Foods. 12:4432. doi: 10.3390/foods12244432 38137236 PMC10742561

[ref32] Xue T. Lu Y. Yang H. Hu X. Zhang K. Ren Y.. (2022). Isothermal RNA amplification for the detection of viable pathogenic Bacteria to estimate the Salmonella virulence for causing enteritis. J. Agric. Food Chem. 70, 1670–1678. doi: 10.1021/acs.jafc.1c07182, PMID: 35099949

[ref33] Zhang M. Liu C. Shi Y. Wu J. Wu J. Chen H. (2020). Selective endpoint visualized detection of *Vibrio parahaemolyticus* with CRISPR/Cas12a assisted PCR using thermal cycler for on-site application. Talanta 214:120818. doi: 10.1016/j.talanta.2020.120818, PMID: 32278427

[ref34] Zuo X. Fan C. Chen H. Y. (2017). Biosensing: CRISPR-powered diagnostics. Nat. Biomed. Eng. 1, 1–2. doi: 10.1038/s41551-017-0091

