# Supplementary figures and images for: A rapid and naked-eye methicillin resistant Staphylococcus aureus screening method based on CRISPR/Cas12a and hybridization chain reaction
Source: Front Microbiol. 2025 Jul 16;16:1592153. doi: 10.3389/fmicb.2025.1592153 (PMC12309411; doi:10.3389/fmicb.2025.1592153)

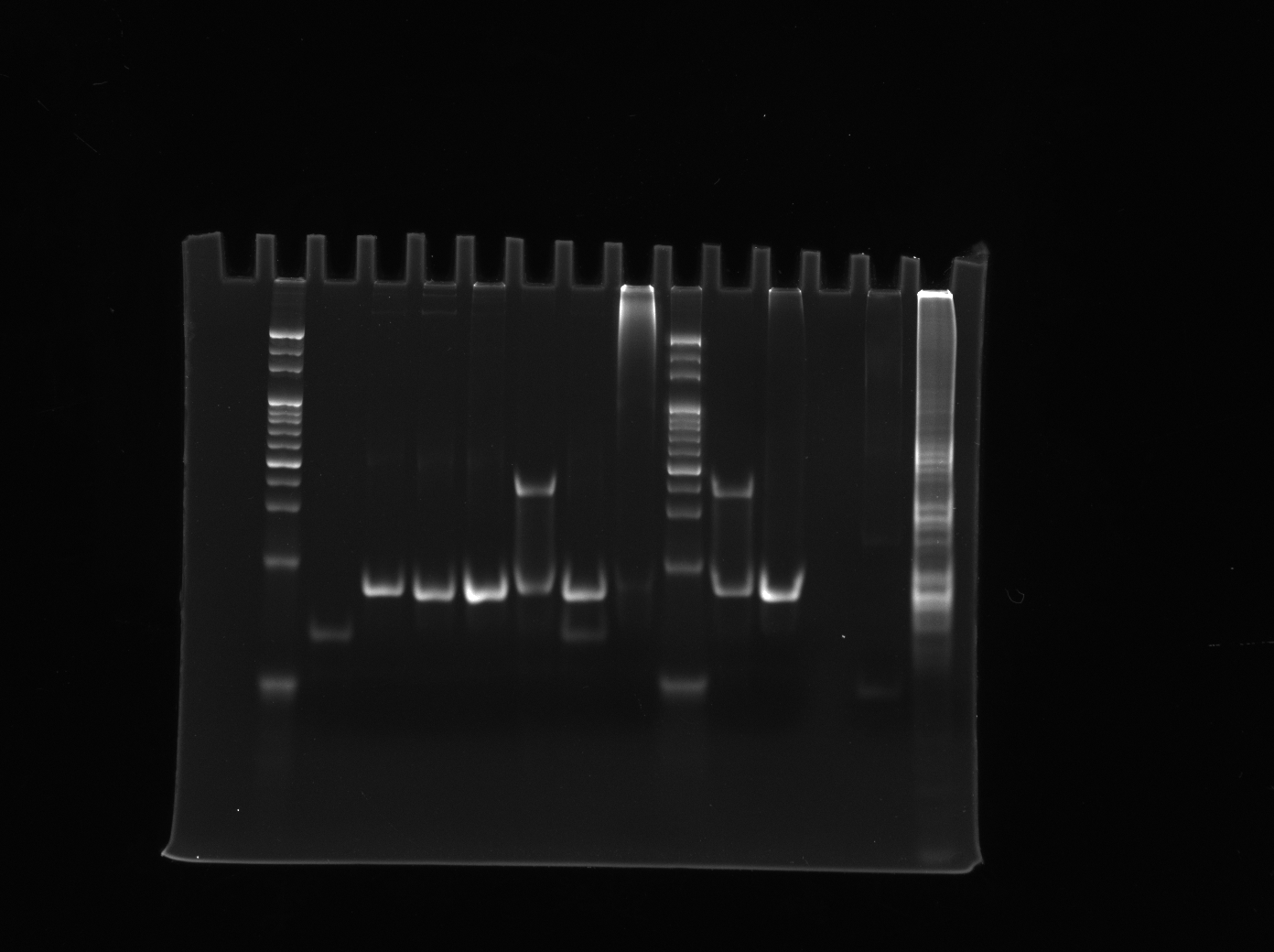

Supplement: Supplementary file 1 [file Image_1.tif]
