# Supplementary material for: A rapid and naked-eye methicillin resistant Staphylococcus aureus screening method based on CRISPR/Cas12a and hybridization chain reaction
Source: Front Microbiol. 2025 Jul 16;16:1592153. doi: 10.3389/fmicb.2025.1592153 (PMC12309411; doi:10.3389/fmicb.2025.1592153)
Supplement: Supplementary file 2 [file Supplementary_file_1.docx]

**A Rapid and Naked-eye Methicillin Resistant Staphylococcus Aureus Screening Method Based on CRISPR/Cas12a and Hybridization Chain Reaction**

**Yayun Jiang****^1*^, Zongyao Chen^3^, Xiao Liu^3^, Qi xin^3^, Dengchao Wang^3^, Caixia Ji^3^, Youwei Li^2*^, Gang Mai^2*^**

^1^ Digestive Diseases Center, Deyang People's Hospital Affiliated to Chengdu University of Traditional Chinese Medicine, Deyang, Sichuan, China.

^2^ Department of General Surgery (Hepatopancreatobiliary Surgery), Deyang People's Hospital Affiliated to Chengdu University of Traditional Chinese Medicine, Deyang, Sichuan, China.

^3^ Department of Clinical Laboratory, Deyang People's Hospital Affiliated to Chengdu University of Traditional Chinese Medicine, Deyang, Sichuan, China.

^*^Corresponding authors: dyjiangyayun@163.com (Yayun Jiang) and maigang68@hotmail.com (Gang Mai).


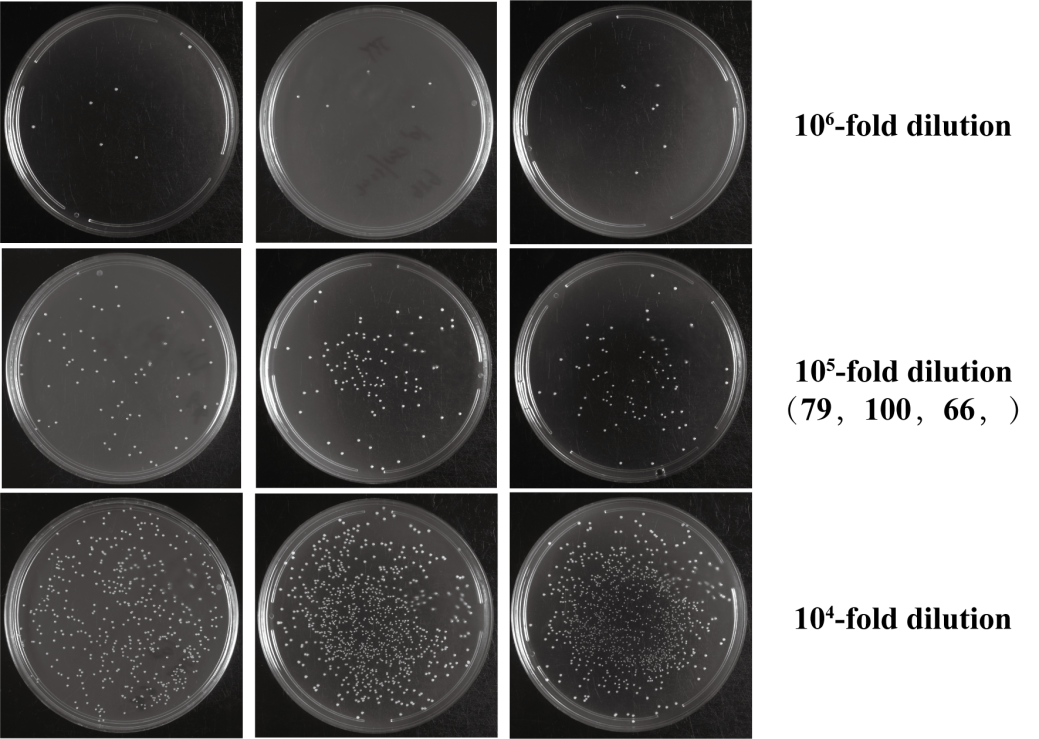


**Fig. S1 Calculation of colony concentration by plate count method.**

The optical density of bacterial suspension is adjusted to absorbance at 600 nm is 1.0. The bacterial suspension is continuously diluted, and 10 μL of bacterial solution diluted 10⁴ to 10⁶ times was pipetted and evenly spread onto agar plates.

**Table S1.** The sequences of oligonucleotides are used in this work.

| **Names** | **Sequences (5’-3’)** |
| --- | --- |
| H0 | ACCTTAATCTGACAACAACTACCAGCTTATCCCA |
| H1 | CTGACAACAACTACCAGCTTATCCCACCTTAATGGGATAAGCTGGTAGTTGTTGTCAGATTAAGGT |
| H2 | TTAAGGTGGGATAAGCTGGTAGTTGTTGTCAGACCTTAATCTGACAACAACTACCAGCTTATCCCA |
| Biotin-H0 | TTTTTTTTTTTTACCTTAATCTGACAACAACTACCAGCTTATCCCA |
| H1-FQ | CTGACAACAAC/i6FAMdT/ACCAGCTTATCCCACCTTAATGGGATAAGCTGG/iBHQ1dT/AGTTGTTGTCAGATTAAGGT |
|  |  |

**Table S2.** The sequences of crRNA and primer are used in this work.

| **Names** | **Sequences (5’-3’)** |
| --- | --- |
| crRNA1 | UAAUUUCUACUAAGUGUAGAUUGAAGACUAUAUCAAACAAC |
| crRNA2 | UAAUUUCUACUAAGUGUAGAUAGUGGAACGAAGGUAUCAUC |
| crRNA3 | UAAUUUCUACUAAGUGUAGAUCUAGAGGAUAGUUACGACUU |
| RPA-F(*mecA*) | GCAATCGCTAAAGAACTA |
| RPA-R(*mecA*) | TGGGACCAACATAACCTA |

**Table S3** The information for clinically isolated strains of *S. aureus* and screening for methicillin resistance by Vitek 2 system using oxacillin MIC method.

| Sample number | Specimen type | MIC of oxacillin ^a^ | Cefoxitin screening test | Susceptibility testing |
| --- | --- | --- | --- | --- |
| S1 | Pus | <=0.25 | Neg | MSSA |
| S2 | Sputum | 0.5 | Neg | MSSA |
| S3 | Secretion | 0.5 | Neg | MSSA |
| S4 | Pus | 0.5 | Neg | MSSA |
| S5 | Secretion | <=0.25 | Neg | MSSA |
| S6 | Pus | 0.5 | Neg | MSSA |
| S7 | Pus | 0.5 | Neg | MSSA |
| S8 | Abdominal fluid | 0.5 | Neg | MSSA |
| S9 | Sputum | 0.5 | Neg | MSSA |
| S10 | Sputum | <=0.25 | Neg | MSSA |
| S11 | Sputum | 0.5 | Neg | MSSA |
| S12 | Sputum | 0.5 | Neg | MSSA |
| S13 | Sputum | <=0.25 | Neg | MSSA |
| S14 | Sputum | 0.5 | Neg | MSSA |
| S15 | Pus | 0.5 | Neg | MSSA |
| S16 | Sputum | <=0.25 | Neg | MSSA |
| S17 | Sputum | <=0.25 | Neg | MSSA |
| S18 | Pus | <=0.25 | Neg | MSSA |
| S19 | Sputum | 0.5 | Neg | MSSA |
| S20 | Sputum | 0.5 | Neg | MSSA |
| S21 | Secretion | 0.5 | Neg | MSSA |
| S22 | Urine | 0.5 | Neg | MSSA |
| S23 | Sputum | 0.5 | Neg | MSSA |
| S24 | Secretion | 0.5 | Neg | MSSA |
| S25 | Sputum | <=0.25 | Neg | MSSA |
| S26 | Sputum | <=0.25 | Neg | MSSA |
| S27 | Sputum | <=0.25 | Neg | MSSA |
| S28 | Sputum | 0.5 | Neg | MSSA |
| S29 | Sputum | <=0.25 | Neg | MSSA |
| S30 | Secretion | <=0.25 | Neg | MSSA |
| S31 | Sputum | >=4 | Pos | MRSA |
| S32 | Sputum | >=4 | Pos | MRSA |
| S33 | Pus | >=4 | Pos | MRSA |
| S34 | Secretion | >=4 | Pos | MRSA |
| S35 | Blood | >=4 | Pos | MRSA |
| S36 | Sputum | >=4 | Pos | MRSA |
| S37 | Sputum | >=4 | Pos | MRSA |
| S38 | Secretion | >=4 | Pos | MRSA |
| S39 | Sputum | >=4 | Pos | MRSA |
| S40 | Secretion | >=4 | Pos | MRSA |
| S41 | Secretion | >=4 | Pos | MRSA |
| S42 | Secretion | 1 | Pos | MRSA |
| S43 | Sputum | >=4 | Pos | MRSA |
| S44 | Broncho-alveolar lavage | >=4 | Pos | MRSA |
| S45 | Sputum | >=4 | Pos | MRSA |
| S46 | Secretion | >=4 | Pos | MRSA |
| S47 | Broncho-alveolar lavage | >=4 | Pos | MRSA |
| S48 | Sputum | >=4 | Pos | MRSA |
| S49 | Secretion | >=4 | Pos | MRSA |
| S50 | Sputum | >=4 | Pos | MRSA |
| S51 | Broncho-alveolar lavage | >=4 | Pos | MRSA |
| S52 | Urine | >=4 | Pos | MRSA |
| S53 | Sputum | >=4 | Pos | MRSA |
| S54 | Abdominal fluid | >=4 | Pos | MRSA |
| S55 | Secretion | >=4 | Pos | MRSA |
| S56 | Secretion | >=4 | Pos | MRSA |
| S57 | Secretion | >=4 | Pos | MRSA |
| S58 | Secretion | >=4 | Pos | MRSA |
| S59 | Secretion | >=4 | Pos | MRSA |
| S60 | Secretion | >=4 | Pos | MRSA |

^a^, The determination of methicillin resistance of *S. aureus* is completed by the Vitek 2 system using the oxacillin MIC method (oxacillin concentrations range from 0.25 to 4 μg/mL). According to the CLSI drug susceptibility standard ^[1]^, *S. aureus* with a MIC of oxacillin ≥4 μg/mL is MRSA, while a MIC of oxacillin ≤2 μg/mL is MSSA.

[1] CLSI. CLSI M100-ED29: 2021 Performance Standards for Antimicrobial Susceptibility Testing, 30th Edition. Clsi vol. 40 (2020).


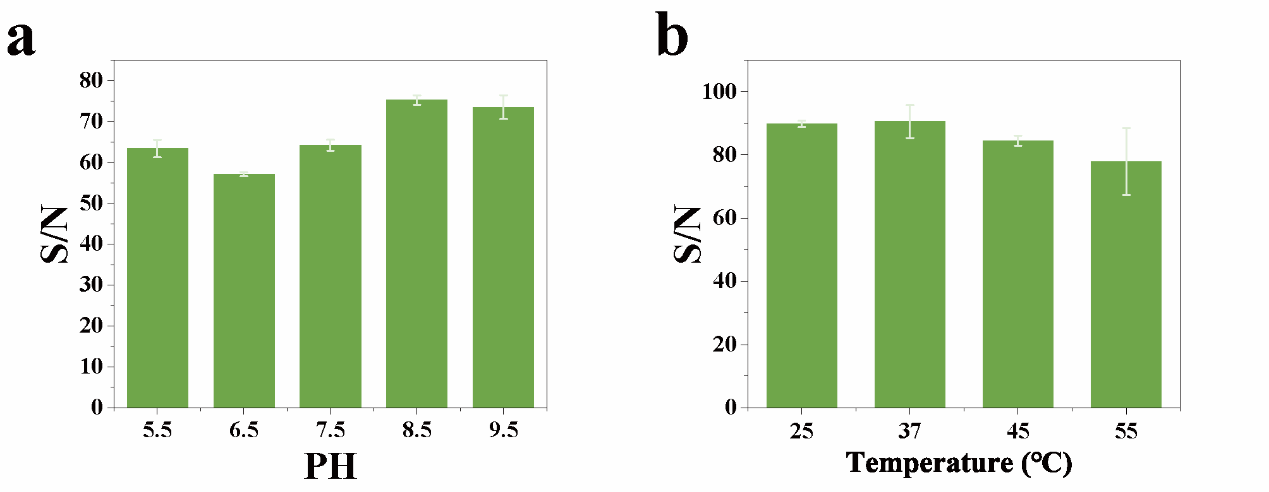


**Fig. S2** Optimization of the reaction parameters. (a) The S/N ratio of fluorescence signals at different PH of HCR. (b) The S/N ratio of fluorescence signals at different temperatures of HCR.


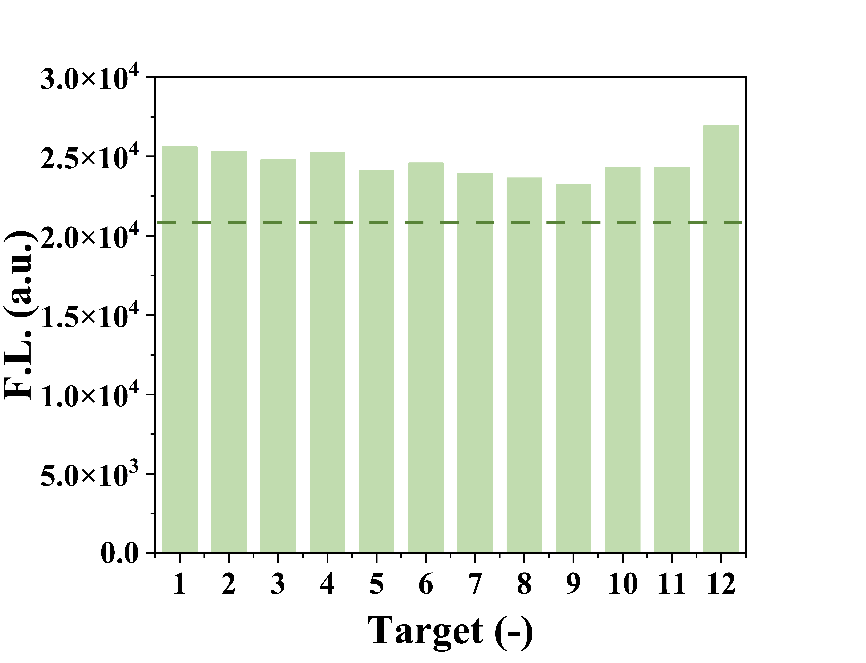


**Fig. S3** Calculate the cut-off value for fluorescent outputs of the MCFHCR. X + 3SD calculates the cut-off value, X represents an average signal value of three replicates without using the target gene, and SD represents three standard deviations.


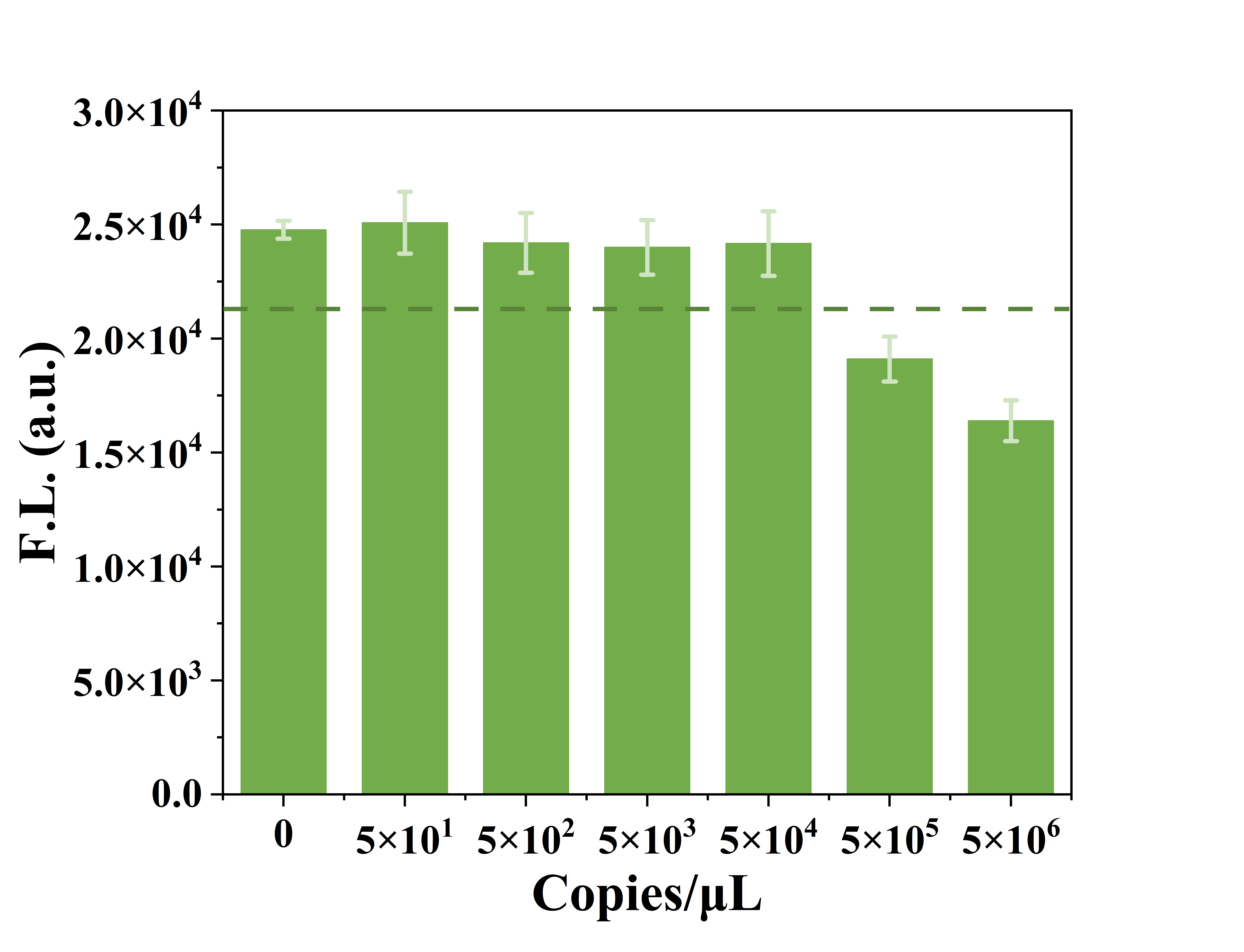


**Fig. S4** Sensitivity analysis of the MCFHCR method by different concentrations of *mecA*, ranging from 0 to 5×10^6^ copies/μL. Error bars represent mean ± SD, where n = 3 replicates.


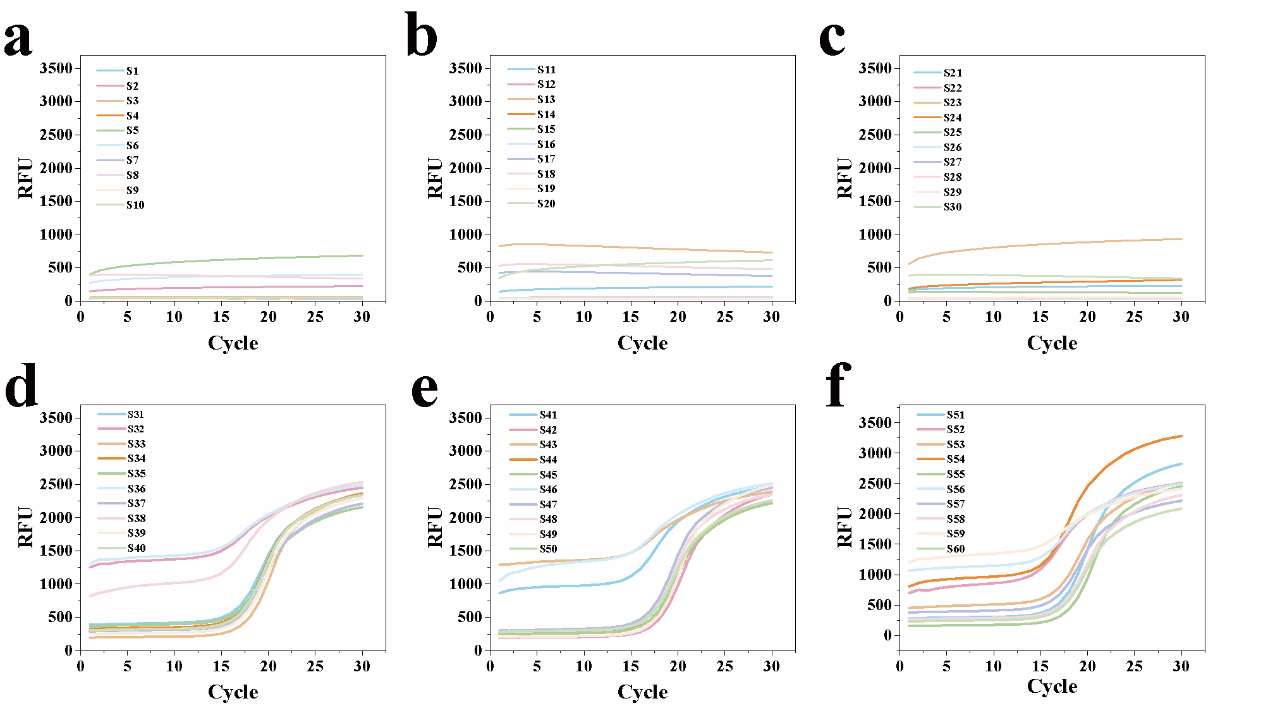


**Fig. S5** qPCR for the detection of clinical samples.


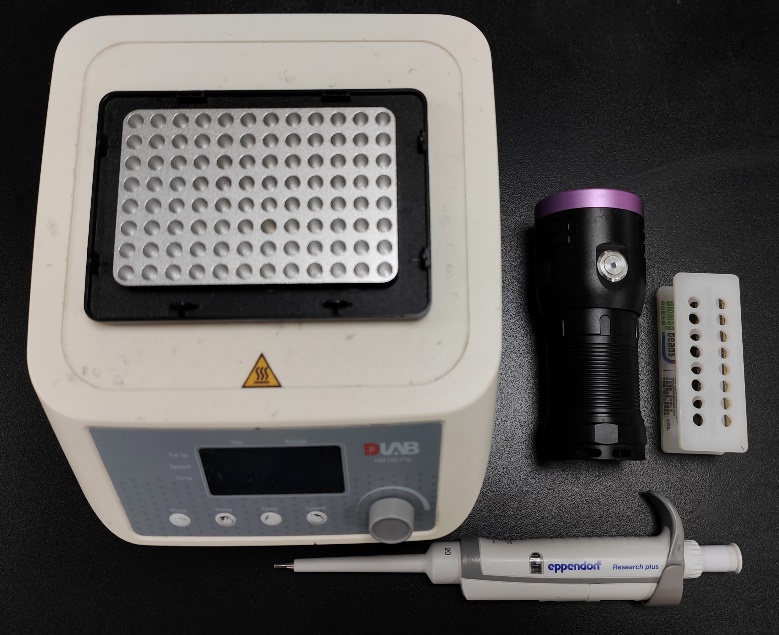


**Fig. S6** The instruments required for the MCFHCR method.
